# Diversity, equity, and inclusion as scientific rigor: progress during challenging times

**DOI:** 10.1093/jncics/pkag046

**Published:** 2026-06-08

**Authors:** Leticia M Nogueira, Matthew F Hudson, Barbara Pistilli, Howard Hochster, Stefan Ambs, Martin R Stockler, James B Yu, Eduardo L Franco

**Affiliations:** Surveillance, Prevention, and Health Services Research, American Cancer Society, Atlanta, GA, United States; Prisma Health, University of South Carolina School of Medicine, Greenville, SC, United States; Department of Medical Oncology, Gustave Roussy, Villejuif, France; IHU PRISM National PRecISion Medecine Center in Oncology, Gustave Roussy, Villejuif, France; Rutgers Cancer Institute, RWJ Barnabas Health, New Brunswick, NJ, United States; Laboratory of Human Carcinogenesis, Center for Cancer Research, National Cancer Institute, Bethesda, MD, United States; NHMRC Clinical Trials Centre, University of Sydney, New South Wales, Australia; Radiation Oncology and Applied Sciences, Dartmouth Hitchcock Medical Center, Lebanon, NH, United States; Division of Cancer Epidemiology, McGill University, Montreal, Canada

High-quality research depends on the questions we ask, the methods we apply, and the perspectives we use to interpret results. Principles related to Diversity, Equity, and Inclusion (DEI) directly shape each of these dimensions. Diverse work environments are better equipped to identify blind spots, interrogate and challenge dominant assumptions, and produce innovative and impactful knowledge.^1-4^ We remain committed to these principles.

Over the past several years, the *Journal of the National Cancer Institute (JNCI), JNCI Cancer Spectrum*, and *JNCI Monographs* undertook a series of deliberate efforts to advance DEI across editorial processes and scholarly output. These efforts reflect a growing recognition that excellence in cancer research is inseparable from DEI principles,^2^ encompassing whose work is published,^3^ as well as how scientific questions and findings are framed, analyzed, and interpreted.^5^

In 2022, Ganz et al. articulated foundational DEI commitments for the Journals, including improving accessibility of the submission process, diversity across the editorial board and reviewer pool, and use of bias-free, inclusive, and precise scientific language.^6^^,^^7^ The use of inclusive language is a critical component of scientific research because language shapes how research questions are framed and how findings are interpreted and applied. Consistent use of inclusive terminology aligned with current scientific understanding helps reduce the risk of reinforcing stereotypes, deficit-based narratives, and/or historically rooted scientific biases and blind spots.

In 2023, Yabroff et al. described early implementation of DEI principles, including adoption of Editor term limits as a structural mechanism to avoid stagnation in perspectives and decision making, and to help diversify the breadth and depth of expertise among editorial board members. Additionally, the editorial introduced specific recommendations for research, including using language, hypotheses, and interpretation of findings that center the conditions imposed on individuals from different sociodemographic groups.^8^^,^^9^

After these publications, DEI efforts continued to expand. For example, prospective authors may now more easily access detailed inclusive language guidelines,^10^ and the Journals recently introduced several new DEI-relevant subject terms for article classifications. Additionally, *JNCI* introduced a dedicated DEI review process to systematically evaluate submissions for alignment with DEI best practices. The Journals are collaborating to refine and standardize the DEI review process, as well as providing training materials for the Editorial Boards.

Together, these efforts aim to improve both the quality and diversity of scientific publications across the Journals by broadening the range of perspectives, topics, and methodological approaches represented. The Journals’ Editorial Boards remain committed to advancing DEI principles and encourage the field toward scholarly discourse on best practices in every aspect of the peer-review process.

## Enhancing rigor, relevance, and reach through implementation of DEI principles

Diverse perspectives have long been recognized as central to producing high-quality research that is relevant across populations and settings.^1-4^ The DEI initiatives at *JNCI, JNCI Monographs,* and *JNCI Cancer Spectrum* supported a more globally representative evidence base among published articles, which not only helped advance DEI-relevant scientific knowledge,^11-15^ but also enhanced the novelty and generalizability of scientific knowledge relevant to all populations.^16-19^

As noted by Yabroff et al. and explained in the Journals’ Detailed Inclusive Language Guidelines,^8-10^ race and ethnicity are social constructs with no biological meaning,^5^ and conflating these social constructs with genetic ancestry is harmful.^20^^,^^21^ Therefore, the Journals continue to recommend using analytical frameworks that contribute to dismantling structural racism in cancer research,^22^ such as identifying the modifiable social, environmental, and structural factors contributing to observed and persistent disparities^23^ and reducing their impact on the entire population, rather than simply reporting the statistical significance of associations and disparities between racialized groups.

By examining the role of modifiable factors contributing to observed disparities,^24^^,^^25^ recently published studies that implemented DEI-aligned methodological approaches provided new insight into the structures, mechanisms, and pathways through which past legacies and current realities shape racialized inequities.^26^^,^^27^ Moreover, by focusing on modifiable factors, recent studies also identified leverage points with potential to improve outcomes across the entire population, including behavioral,^28^^,^^29^ environmental (eg, air pollution, climate change),^30^^,^^31^ social,^32^^,^^33^ policy (eg, government-sponsored residential lending, health insurance eligibility and benefits),^34-36^ and health-care system strategies.^24^^,^^25^ Finally, studies evaluating shortcomings in clinical trial geographic availability and eligibility criteria, which are also modifiable, contributed to new evidence base strategies that are aimed at improving access to novel therapies and technologies for the entire population.^37-39^

The Journals’ adoption of DEI principles also encouraged more productive and nuanced discussions of social, cultural, and structural factors that shape the relevance and feasibility of proposed cancer control strategies and interventions.^40^^,^^41^ Collectively, these discussions emphasize the importance of developing research questions, strategies, and interventions in partnership with the communities who have relevant lived experiences and vital subject-matter expertise.^42-45^ Moreover, the intentional inclusion of diverse perspectives, lived experiences, and rigorous methodological approaches also facilitates transdisciplinary engagement^46^ and encourages teams to scrutinize long-held assumptions, data limitations, and sources of uncertainty.^24^^,^^25^^,^^47-49^

As studies increasingly quantify the relative contribution of various individual, socioeconomic, and structural factors to measured disparities, the persistent residual disparity in cancer outcomes suggests important limitations in the prevailing conceptual frameworks and measures used in cancer research.^24^^,^^36^^,^^50^^,^^51^ Therefore, expanding the scope of cancer research to incorporate concepts, methods, and measures that are well established in adjacent scientific fields is vital for continued scientific progress in cancer research.^52^ Illustrative examples of this form of intellectual courage include a critical reflection on the legacy of colonialism on structural determinants of tobacco-related cancer disparities^49^^,^^53^^,^^54^ and an article evaluating the influence of political determinants of cancer outcomes.^33^

In summary, by affirming DEI as core editorial principles, *JNCI, JNCI Monographs*, and *JNCI Cancer Spectrum* demonstrate a commitment to creating scholarly spaces where critical questions can be asked, supporting a climate of respectful deliberation where assumptions can be examined, and intentionally inviting historically excluded expertise to inform the evidence base. This ongoing commitment to advancing diversity, equity, and inclusion across every aspect of peer review and post-publication scholarly discussion is an active investment in a more just, innovative, and impactful scientific enterprise.

## Data Availability

No new data were generated or analyzed for this editorial.
